# Development and Characterization of Eudragit-RL-100-Based Aceclofenac Sustained-Release Matrix Pellets Prepared via Extrusion/Spheronization

**DOI:** 10.3390/polym13224034

**Published:** 2021-11-21

**Authors:** Mohamed Abbas Ibrahim, Doaa Hasan Alshora

**Affiliations:** Department of Pharmaceutics, College of Pharmacy, King Saud University, Riyadh 11451, Saudi Arabia; dalahora@ksu.edu.sa

**Keywords:** aceclofenac, Eudragit RL 100, sustained-release matrix pellets, optimization, extrusion/spheronization

## Abstract

Aceclofenac (AC) is a nonsteroidal anti-inflammatory drug used in the treatment of chronic pain in conditions such as rheumatoid arthritis, with frequent administration during the day. The formulation of sustained release matrix pellets can provide a promising alternative dosage form that controls the release of the drug, with less blood fluctuation and side effects—especially those related to the gastric system. The extrusion/spheronization technique was used to formulate AC matrix pellets. The response surface methodology (version 17.2.02.; Statgraphics Centurion) was used to study the impacts of Eudragit RL 100 and PVP K90 binder solution concentrations on the pellets’ wet mass peak torque, pellet size, and the release of the drug. Statistically, a significant synergistic effect of PVP K90 concentration on the peak torque and pellet size was observed (*p* = 0.0156 and 0.031, respectively), while Eudragit RL 100 showed significant antagonistic effects (*p* = 0.042 and 0.013, respectively). The peak torque decreased from 0.513 ± 0.022 to 0.41 ± 0.021 when increasing the Eudragit RL 100 from 0 to 20%, and the pellet size decreased from 0.914 ± 0.047 to 0.789 ± 0.074 nm. The tested independent factors did not significantly affect the drug release in the acidic medium within 2 h, but these pellet formulae maintained the drug release at less than 10% in the acidic medium (pH 1.2), which may decrease gastric irritation side effects. In contrast, a highly significant synergistic effect of Eudragit and highly antagonistic effect of the PVP solution on drug release in the alkaline-pH medium were observed (*p* = 0.002 and 0.007, respectively). The optimized pellet formula derived from the statistical program, composed of 3.21% Eudragit and 5% PVP solution, showed peak torque of 0.861 ± 0.056 Nm and pellet size of 1090 ± 85 µm, and resulted in a significant retardation effect on the release after 8 h compared to the untreated drug.

## 1. Introduction

Aceclofenac (AC) is a non-steroidal anti-inflammatory drug (NSAID), and it is prescribed and recommended in the treatment of osteoarthritis and rheumatoid arthritis to relieve pain. Moreover, AC has the ability to reduce morning stiffness and improve spine movement in a comparable manner to indomethacin and tenoxicam. It acts by blocking the effects of natural substances called cyclooxygenase (COX) enzymes, which act as the key step in the formation of prostaglandin 2, which plays a critical role in inflammation [1,2]. AC is used to relieve chronic pain, and this requires frequent administration of the drug for a long time. A dosage regimen of 100 mg twice daily for up to 6 months is recommended for the treatment of a patient with osteoarthritis of the knee [3]. The most common adverse effects of AC are related to the GI systems, as with other NSAIDs, including diarrhea, flatulence, gastritis, constipation, vomiting, and ulcerative stomatitis, with a frequency rate of <5%. Dyspepsia and abdominal pain can also occur, but at a higher rate than 5% [4]. Several clinical trials have shown similar GI event rates after the administration of AC and other NSAIDs: 8–28% after AC, and 15–36% after other NSAIDs, including indomethacin, diclofenac, piroxicam, ketoprofen, tenoxicam, and naproxen [5,6,7,8]. From a pharmacokinetic point of view, AC bioavailability is low, with an elimination half-life of 4 h [2]. 

Sustained-release formulation provides several advantages over conventional oral dosage forms; it is formulated to stabilize the drug concentration in the blood and decrease its fluctuation. Moreover, it is formulated to minimize the side effects of the drug. In some cases, continuous and prolonged release of the drug will be effective in achieving a certain therapeutic effect, due to its short half-life and low bioavailability. This is the main target in the treatment of chronic diseases such as osteoarthritis or rheumatoid arthritis. Different research articles have formulated AC as a sustained-release dosage form. The formulation of AC sustained-release matrix tablets with different grades of hydroxypropyl methylcellulose (HPMC)—with or without PVP K30—has been carried out by Ankita et al. [9]; all formulations successfully sustained the release for up to 24 h [9]. Polymer-based sustained release of microspheres was prepared using rosin polymer, with polyvinyl alcohol as an emulsifying agent [10]; only 55% of the drug was released over 24 h, due to the hydrophobic nature of the polymer [10]. Ghosh et al. [11] formulated AC SR matrix tablets using different grades of HPMC, ethylcellulose, and guar gum; it was found that HPMC K4 provides a reliable sustained effect, with good stability compared to freshly prepared tablets [11].

Multiparticulate oral solid dosage forms or pellets are spherical in shape, with sizes ranging from 500 to 1500 µm. Pellet formulations have attracted researchers’ attention, since they provide several advantages over conventional solid dosage forms. The large surface area of the pellets is considered to be a major advantage of this formulation. Once administered, the pellets will be distributed over the gastrointestinal tract (GIT), with a low risk of gastric irritation. Pellets can be produced using different techniques, including the powder-layering technique [12], solution-/suspension-layering technique [13], extrusion/spheronization technique [14,15], balling/spherical agglomeration [16], spray-congealing/drying [16], cryopelletization [16], and melt spheronization [16].

Coating of the pellets with polymer film has been employed by several researchers to control the release of drugs. Budesonide SR coated pellets with Eudragit were prepared by Raval et al. [17]; the results showed the efficacy of Eudragit S100 not only in sustaining the release of the drug, but also in decreasing the intensity of gastric irritation by preventing the release in the first 2 h [17]. Coated pellets containing salbutamol were prepared using a combination of different Eudragit types (RSOP and L100); the resultant pellets provide a good sustained release over 8 h [18]. There are few studies concerning the use of Eudragit as a matrix former in matrix pellets. For example, É. Bölcskei et al. [19] prepared immediate-release matrix pellets containing diclofenac sodium, based on Eudragit NE 30D as a binder and matrix former, via extrusion/spheronization procedures; they studied the critical material and process parameters that control pellet attributes and drug dissolution by means of a factorial design. Furthermore, Amin et al. [20] prepared SR matrix pellets containing lornoxicam via extrusion/spheronization, using a 3^2^ full factorial design to study the effects of Eudragit RLPO and Eudragit RSPO on drug release rates. 

Technically, coating is a complex and time-consuming process. It requires the preparation of some organic solvents, and these solvents may present some health hazards. Moreover, the release of a drug from coated dosage forms mainly depends on the thickness of the coating, which plays a crucial role in controlling the release. Therefore, matrix pellets provide several advantages over coated pellets, such as eliminating the use of explosive solvents, as well as simplifying the process. Extrusion/spheronization procedures are currently among the methods utilized to manufacture pharmaceutical pellets. The characteristics and properties of the manufactured pellet formulations can be manipulated by controlling both materials’ composition and extrusion/spheronization conditions. It is worth mentioning that the formulation of AC sustained-release matrix pellets has not been discussed in the literature. In our study, sustained-release matrix pellets containing AC were manufactured without the use of coating procedures. The formulation of AC SR matrix may might provide several advantages over the use of coating procedures for SR purposes, as mentioned previously, in addition to controlling the drug release from the formulated pellets by controlling pellet wet mass properties. Formulations of AC as SR matrix pellets are expected to be able to provide and maintain therapeutically effective plasma concentrations for a period longer than the untreated drug after oral administration. In this current study, Eudragit RL 100 was used as a polymer and PVP K90 as a mass-forming agent to sustain the release of AC. A 3^2^ full factorial design was used to determine the effects of different concentrations of Eudragit RL100 (X1) and PVP K90 (X2) on different response parameters, including mean line torque (Y1), pellet size (Y2), % drug released after 2 h (Y3), and % drug released after 8 h (Y4).

## 2. Materials and Methods 

AC was purchased from Sigma-Aldrich (Saint Louis, MI, USA). Microcrystalline cellulose (MCC; Avicel^®^ PH101) was obtained from Serva Feinbiochemica (Heidelberg, Germany). BASF (Geismar, LA, USA) supplied polyvinylpyrrolidone (Kollidon^®^, PVP K90). Evonik-Degussa GmbH. (Essen, Germany) kindly donated Eudragit RL 100. Other materials and solvents did not require further purification, and were of reagent or analytical grade.

### 2.1. Experimental Design

The influences of two independent factors (Eudragit RL 100 (X1) and PVP binder solution (X2)) on the characteristics of AC SR matrix pellets using the 3^2^ full factorial design were evaluated. Statistical analysis was conducted using Statgraphics software (version 17.2.02.; Statgraphics Centurion). Statistical models (individual, interactive, and quadratic effects) were analyzed in order to assess the impacts of the tested independent variables on the characteristics of the pellets (responses), viz., wet mass peak torque (Nm, Y1), pellet size (µm, Y2), AC release at pH 1.2 after 2 h (Y3), and AC release at pH 7.4 after 8 h (Y4). Table 1 illustrates the levels of the tested independent variables. 

### 2.2. Assessment of Pellet Wet Mass (Mixer Torque Rheometry (MTR)) 

The wet masses of the tested pellet formulae were measured via MTR using an MTR-3 mixer torque rheometer (Caleva, Dorset, UK), prior to extrusion/spheronization, in order to calculate the PVP solution volume (binder ratio) needed for maximum wet mass peak torque. The measurement was carried out in a stainless steel vessel (135 mL capacity) with two knife-edge mixing blades attached, which was adjusted at a speed of 50 rpm. Twenty grams of the excipients’ powders were mixed using a Turbula mixer (Erweka type S27; Apparatebau, Germany) for 10 min, and then added to the MTR vessel. Aqueous PVP binder solutions were prepared by dissolving the required amount of PVP in distilled water. Thereafter, 5 mL of binder liquid (PVP solution) was added to the vessel over 5 intervals for wet massing. Each wet massing measuring cycle involved 60 s for mixing and 20 s for data logging (gathering). During the wet massing procedures, the consistency of pellet wet mass (represented by the mean line torque; Nm) was computed [21]. Moreover, the binder ratio (mL of PVP binder solution required for 1 g of powder to attain wet mass peak torque) was determined. The acquisition and curation of the obtained data were achieved using a data acquisition system and software package.

### 2.3. Extrusion/Spheronization Procedures

AC sustained-release matrix pellets containing 15% drug were produced via the extrusion/spheronization method. Aqueous PVP solution (containing different polymer concentrations: 1, 3, and 5%) was utilized as a binder. The ratio of binder PVP solution to powder required for powder wet massing was calculated from the maximum wet mass torque value acquisition based on MTR studies (Table 3). Table 1 displays the compositions of different AC matrix pellet formulations. Powdered excipients (Avicel^®^ PH101, Eudragit RL 100, and the AC) were blended for 10 min in a Turbula mixer and then added to the MTR vessel. The required volume of the PVP solution was added, and the powder was wetted with the binder solution for 10 min. The obtained wet mass was then subjected to extrusion through a screen pore size of 1 mm Ø (Mini Screw Extruder, Model MSE1014; Caleva, Dorset, UK) at an extrusion speed of 100 rpm [22]. Consequently, the produced rod-shaped extrudates were spheronized for 5–7 min at a speed of 700 rpm using a rotating plate of even cross-hatch geometry (Spheronizer Model 120; Caleva, Dorset, UK). The resulting spheroids were finally dried for 5 h at 60–70 °C in a hot oven. 

### 2.4. Drug Content

AC content in the prepared pellets was calculated in triplicate by using UV spectrophotometry. The pellet formula was crushed, 50 mg of which was placed in 250 mL of phosphate buffer (pH 7.4), sonicated for 15 min, and filtered through a 0.45 μm filter (Sartorius, Göttingen, Germany) [23] AC content was measured spectrophotometrically at 276 nm using a calibration curve in phosphate buffer, pH 7.4, in a concentration range of 5–30 µg/mL (UV-2800 spectrophotometer Labomed Inc., Los Angeles, CA, USA).

### 2.5. Particle Size 

The sizes of the manufactured AC SR matrix pellets were measured via laser diffractometer (Mastersizer Scirocco 2000; Malvern Instruments, Grovewood Road, UK). Approximately 500 mg of the manufactured pellet formulation was added to the sample micro feeder and measured five times. The average volume-weighted mean size was determined [24].

### 2.6. In Vitro Release 

The USP dissolution basket method (apparatus I) was used to study the in vitro release of AC from the SR matrix pellet formulas using a dissolution tester (LOGAN Instrument Corp., Somerset, NJ, USA). Drug-loaded pellets equivalent to 100 mg of AC were weighed accurately and added to the dissolution flask. The drug release experiment was carried out in triplicate, and the amount of AC released at predetermined time intervals was measured spectrophotometrically at 276 nm up to 8 h, using calibration curves in both 0.1 N HCl (pH 1.2) and phosphate buffer (pH 7.4). For the in vitro release dissolution studies over a pH range relevant to GIT conditions, 750 mL of 0.1 N HCl (pH 1.2) was added to each of the flasks and equilibrated to 37 ± 0.5 °C. Aliquot samples were withdrawn at time intervals for 2 h, and then the pH was changed to 7.4 by adding 250 mL of 0.2 M trisodium phosphate, and the release experiment was continued for a further 6 h. To determine the sustaining behavior of the optimized formula, the release of untreated AC was also performed under the same experimental conditions as all formulations.

## 3. Results

### 3.1. Effect of Independent Factors on Wet Mass

**Figure 1:**
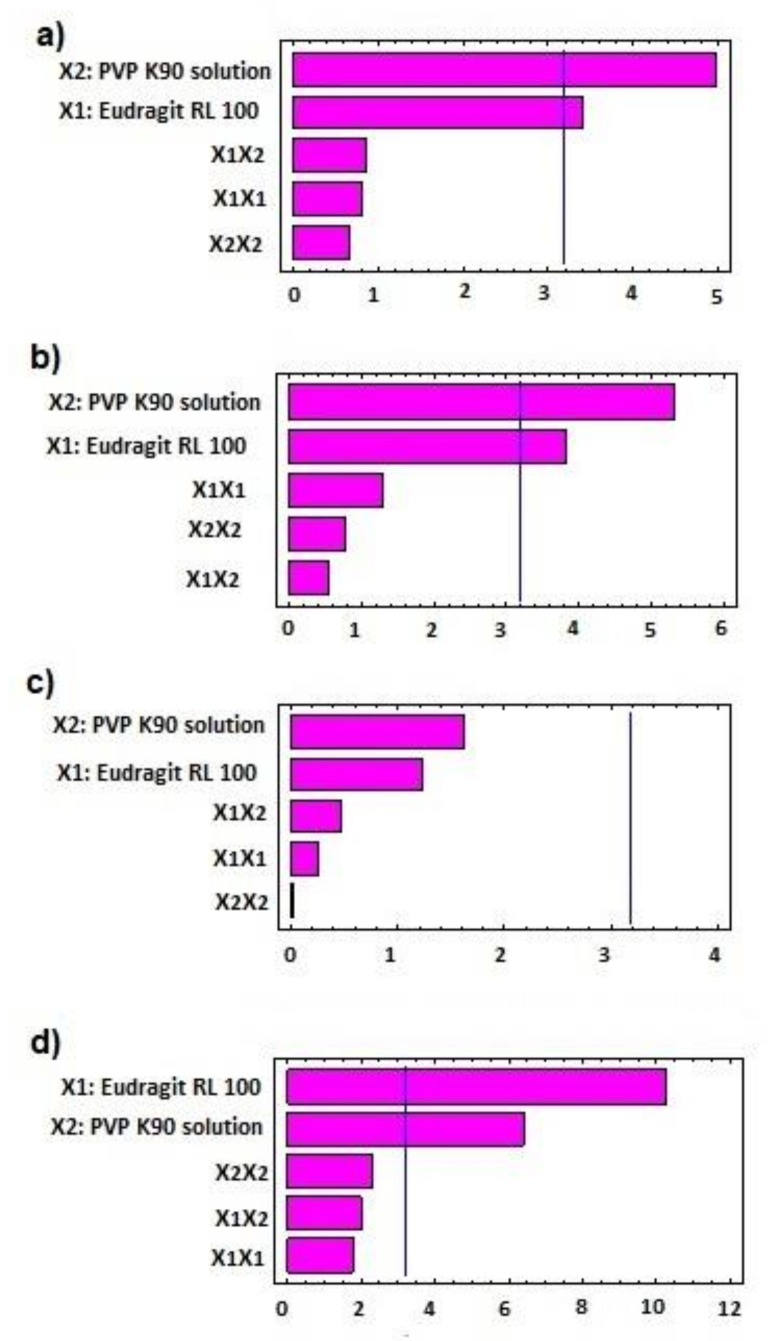
Standardized Pareto chart for (**a**) mean line torque, (**b**) pellet size, (**c**) release after 2 h, and (**d**) release after 8 h of AC sustained-release matrix pellets.

**Figure 2:**
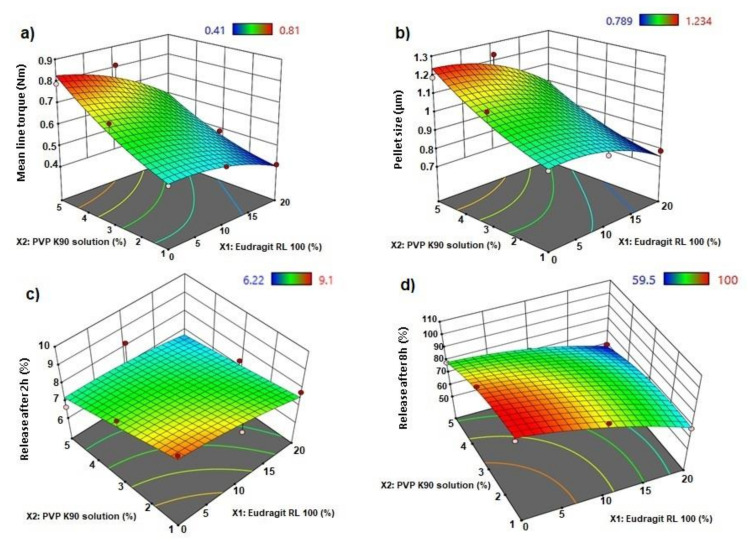
3D response surface plots for (**a**) mean line torque, (**b**) pellet size, (**c**) release after 2 h, and (**d**) release after 8 h of AC sustained-release matrix pellets.

**Figure 3:**
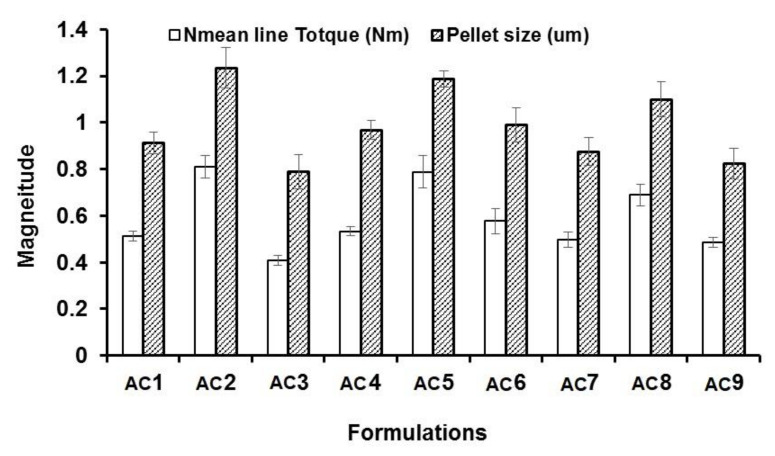
Mean line torque and pellet size of AC from different formulations.

The statistical effects of Eudragit RL 100 (X1) and PVP K90 solution (X2) on the peak torque values of the AC pellets’ wet mass are illustrated in Table 2, as well as the Pareto chart in Figure 1. The ANOVA results depicted in Table 2 indicate that Eudragit exerted a significant antagonistic effect on wet mass peak torque (*p* = 0.042), while PVP solution showed significant synergistic action on pellets’ wet mass peak torque (*p* = 0.016). Only the individual effects of Eudragit and PVP exhibited significant effects on wet mass, while other interactive quadratic effects were found to be insignificant, as displayed in the response surface plot (Figure 2a). It is worth mentioning that the binder ratio (mL of PVP solution required for wet mass peak torque) varied according to the composition of the pellet formula. The binder ratios of the tested wet masses ranged from 0.667 to 0.933 mL/g, as shown in Table 3. As shown in Table 2 and Figure 3, pellet formulae AC2 and AC5 (composed of 10% and 0% Eudragit, respectively, and 5% PVP binder solution) exhibited the greatest peak torque values (0.81 ± 0.047 and 0.789 ± 0.071 Nm, respectively) amongst the tested wet masses. On the other hand, AC3 pellet wet mass showed the lowest peak torque value amongst the formulations. Increasing the concentration of the binder solution (PVP K90) resulted in an increase in the mean line torque of the wet mass. This could be due to the increased cohesiveness of the powder mass and the mean torque line upon increasing the PVP concentration [25]. Additionally, Alshora et al. [15] indicated that increasing the concentration of PVP K30 as a binder resulted in an increase in the wet mass peak torque values of flurbiprofen pellets. Moreover, the level of Eudragit L 100 polymer in the pellet wet mass resulted in a reduction in the peak torque of the wet mass. 

Mahrous [25] found that the extent of peak torque for the Eudragit^®^ systems was lower than that obtained with Avicel^®^ alone, using water as the wet-massing liquid; he attributed this finding to the better interaction of Avicel with the binder via hydrogen bonding.

### 3.2. Drug Content

The AC content in pellet formulae was measured, and the obtained data revealed that drug content ranged from 13.71 ± 0.62 mg (91.4%) to 15.81 ± 0.57 mg (105.40%) of the theoretical content (15 mg), signifying homogeneous drug distribution in the prepared SR matrix pellets (Table 3).

### 3.3. Effect on Pellet Size

The effects of the independent factors (Eudragit and PVP solution) on the size of the sustained-release AC pellets showed the dependence of the pellets’ sizes on the pellet excipients. Eudragit exerted significant antagonism on the pellet size (*p* = 0.031), while the PVP had a synergistic effect on the tested response (*p* = 0.013), as shown in the Pareto chart (Figure 2b) and Table 2. In addition, no statistical significance was observed regarding the interactive and quadratic effects of the excipients on pellet size (*p* < 0.05). The response surface plot in Figure 2b reveals that the particle size of the AC pellets was noticeably increased when increasing the concentrations of PVP binder solution, and decreased when increasing the level of Eudragit in the pellet formulation. The smallest pellet size (789 ± 74 µm) was noted in AC3, in which a low concentration of PVP and high concentration of Eudragit were used. In contrast, the largest pellet size (1230 ± 87 µm) was detected in AC2, which contained a moderate concentration of Eudragit and a high concentration of PVP (Figure 3). The direct relationship between wet mass peak torque and pellet size is consistent with the results obtained by Mahrous et al. [24], who showed that minimizing wet mass mean line torque can cause a noticeable decrease in pellet size. A low pellet wet mass consistency enables easy extrusion of pellets and yields small pellets with smoother surfaces. A similar finding was obtained by Ibrahim and Mahrous [22].

### 3.4. Effect on In Vitro Release 

**Figure 4:**
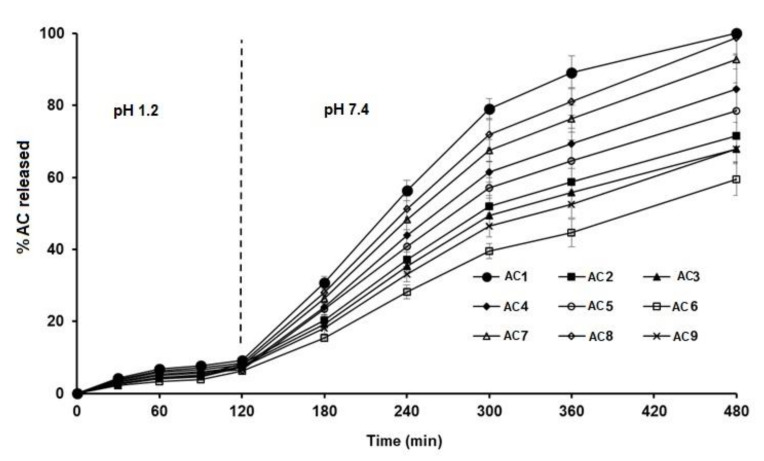
In vitro release profiles of AC from different sustained-release matrix pellet formulations.

The dissolution studies of the AC pellets were performed in 0.1 N HCl for the first 2 h, then shifted to an alkaline pH of 7.4. The dissolution profile (Figure 4) showed that less than 10% of the drug was released within the first 2 h from all formulations. This behavior could be due to the presence of Eudragit, which starts to be ionized at alkaline pH—at which time the release of the drug started to increase [26]—in addition to the acidic nature of AC, which slows its release at low pH values [26]. 

The Pareto chart (Figure 1c) showed that neither Eudragit nor PVP K90 had a significant effect on the release in the first 2 h. Although it was insignificant (*p* > 0.05) (Table 1), the response surface (Figure 2c) showed that at the lowest concentration of Eudragit RL100 and PVP K90 (AC1), the release was highest (9.1%) compared with AC6 (containing the highest concentrations of PVP K90 and Eudragit RL 100), which reduced the release to 6.22 % (Table 3).

By rendering the pH more alkaline, the release of AC sped up, due to the ionization of Eudragit at alkaline pH. At this stage, the Pareto chart (Figure 1d) and the response surface (Figure 2d) showed significant antagonistic effects of Eudragit RL100 (*p* = 0.002) and PVP K90 (*p* = 0.007) (Table 1) on the release. This indicates that increasing the Eudragit concentration to the intermediate point resulted in enhancing AC release from the pellets at low PVP solution concentrations. Formulations (AC1, AC7, AC8) containing 1 and 3% PVP K90 and 0, 10, and 0% Eudragit, respectively, showed the highest release rates amongst the tested formulae (100.00 ± 5.51, 92.78 ± 6.52, and 98.7 ± 4.87, respectively). However, at high Eudragit concentrations (20%), the drug exhibited slow release rates, as was the case for pellet formulae AC3, AC6, and AC9, which contained 20% Eudragit and 1, 5, and 3% PVPB, respectively. The wet mass peak values of these formulations were low, with small particle sizes, as shown in Table 3. At the lowest Eudragit RL 100 concentration, and with increasing the concentration of the binder solution, increasing the mean torque line from 0.513 (AC1) to 0.789 Nm (AC5) dramatically increased the pellets’ size, from 9.14 to 1.187 µm, respectively (Table 3). This increase in the pellet size significantly reduced the release after 8 h, from 100 to 78.52%. A similar finding was obtained by Ibrahim et al. [27], who revealed an inverse relationship between the peak torque values of pellet wet mass and indomethacin release rate from pellet formulations. Moreover, Ibrahim et al. [21] indicated that at a low pellet wet mass, the peak torque was associated with small pellet size and, hence, a rapid drug release rate.

As indicated by the release data, AC showed a biphasic release from the SR matrix pellets. Therefore, the release kinetics of AC from the tested matrix pellet formulae were studied for the two release periods (0–2 h at pH 1.2, and 2–8 h at pH 7.4) using different release kinetics models (Table 4). The release kinetics were determined by the highest correlation coefficient. The results showed that the release of AC from pellets in the first period (acidic pH) followed the zero-order kinetics for formulae F2, F3, F6, and F9 (r^2^ = 0.979), while formulae F1, F4, F5, F7, and F8 followed the Higuchi model, with a correlation coefficient value of 0.998 (Table 4). When the release data were analyzed by calculating the *n* value for the Korsmeyer–Peppas equation, the *n* values ranged from 0.539–0.704, indicating anomalous non-Fickian anomalous release [28]. With respect to AC release at alkaline pH (second phase), the drug followed “zero-order” release kinetics, with a correlation coefficient value 0.975–0.989, which is the highest value compared with other models. The *n* values for all formulations were more than 0.89, supporting super case II transport, where the release is controlled by both diffusion and relaxation of the polymer chain. The super case II relaxational release designates the drug transport process concomitant with stresses and state transition in hydrophilic glassy polymers, and it mainly symbolizes polymeric chain erosion, as in the case of Eudragit RL 100 [28].

### 3.5. Optimization of AC Sustained-Release Matrix Pellet Formulation

**Figure 5:**
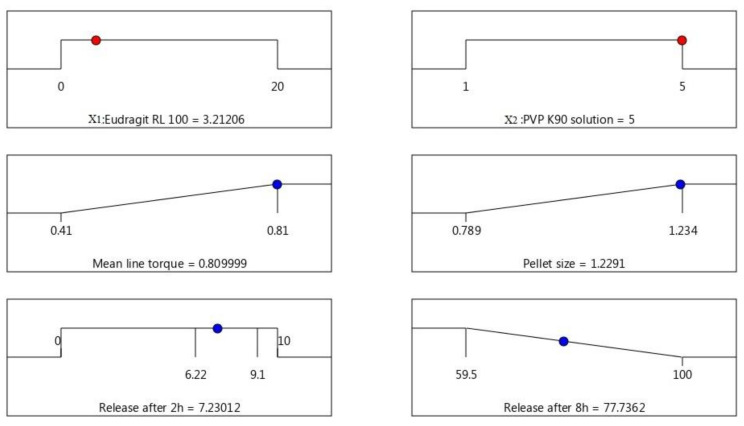
Statistical program checkpoint for the desirability of independent (X1 and X2) and dependent factors of AC sustained-release matrix pellet formulation.

**Figure 6:**
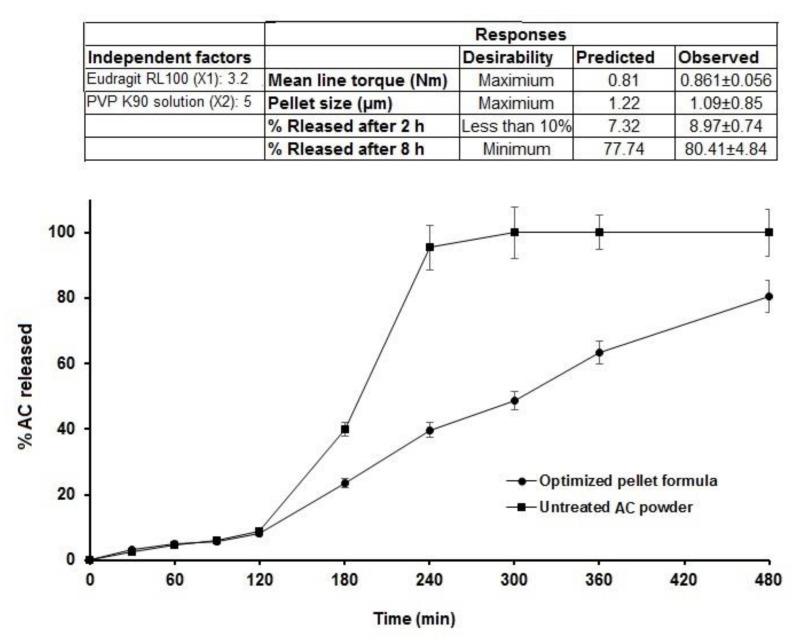
In vitro release profiles of AC from the optimized sustained-release matrix pellet formula in comparison to the untreated drug (the enclosed figure refers to the predicted and observed response values of the optimized pellet formula).

Optimization of AC pellet formulae was established based on the following desirability features: maximum peak torque, maximum pellet size, less than 10% release in the first 2 h (in acidic medium), and minimal release rate after 8 h. A checkpoint of Eudragit RL 100 (X1) = 3.21% and PVP K 90 solution (X2) = 5% was recommended by the statistical program using multiple-response optimization, as shown in Figure 5. The optimized pellet formula was prepared based on the composition suggested by the statistical software, and 0.8 mL of 5% PVP solution/g solid was used as the binder ratio, as obtained from the MTR measurement. The observed values for the attributes (responses) of the sustained-release optimized pellet formula were matched to the predicted responses, and the results revealed a good correlation with the software model’s predicted response values (Figure 6). The optimized pellet formula showed a peak torque of 0.861 ± 0.06 Nm (observed) compared to the predicted value (0.81 Nm), and the observed pellet size was 1090 ± 85 µm, which was narrowly interrelated with the predicted value (1220 µm). In addition, the percentages of drug release in the acidic pH in the first 2 h, and after a further 6 h (at pH 7.4), were 8.97 ± 0.74% and 80.41 ± 4.84%, respectively, showing high correlation with the predicted values (7.32% and 77.74%, respectively).

## 4. Conclusions

Formulations of AC sustained-release matrix pellets successfully controlled the release of the drug, with a minimal amount of drug released in the gastric system, eliminating the GI irritant side effects of the drug. In addition, the results showed controlled in vitro release of AC from the investigated SR pellets. The results show that Eudragit RL 100 exerts significant antagonistic effects on both peak torque and pellet size, while PVP K90 solution exerts significant synergistic effects on them. Moreover, it was noted that increasing the concentration of PVP binder solution significantly retarded the release of the drug after 8 h at alkaline pH.

## Figures and Tables

**Table 1:** Variables and composition in 3^2^ full factorial design for AC-loaded matrix pellets.

Independent Factor				Level					
				Low (−1)		Middle (0)		High (+1)	
X1: Eudragit L100 (%)				0		10		20	
X2: Polyvinylpyrrolidone K90 (PVP) (%)				1		3		5	
Ingredients %	Formula								
	AC1	AC2	AC3	AC4	AC5	AC6	AC7	AC8	AC9
Aceclofenac	15%								
Eudragit L 100	0	10	20	10	0	20	10	0	20
PVP K 90	1	5	1	3	5	5	1	3	3
Avicel PH 101	to 100%								

**Table 2:** Analysis of variance for the impact of Eudragit RL 100 and PVP K90 solution (independent factors) on the properties of AC sustained-release matrix pellet formulations.

Responses	Source	Sum of Square	*p*-Value
Peak Torque	X1: Eudragit RL100	0.04455	0.0422
	X2: PVP K90	0.09526	0.0155
	X1X2	0.00292	0.447
	X1^2^	0.00267	0.4657
	X2^2^	0.00180	0.5425
Pellet Size	X1: Eudragit RL100	0.05980	0.0309
	X2: PVP K90	0.11537	0.0128
	X1X2	0.00130	0.6105
	X1^2^	0.00692	0.2815
	X2^2^	0.00240	0.4964
% Release after 2 h	X1: Eudragit RL100	1.30667	0.3034
	X2: PVP K90	2.25707	0.2019
	X1X2	0.18923	0.6694
	X1^2^	0.05556	0.8148
	X2^2^	0.00109	0.9737
% Release after 8 h	X1: Eudragit RL100	105.15	0.0020
	X2: PVP K90	40.94	0.0077
	X1X2	42.9025	0.1386
	X1^2^	34.445	0.1702
	X2^2^	55.8625	0.1061

**Table 3:** Properties of AC sustained-release matrix pellet formulations.

#	Dependent Factors (Responses) Mean ± Standard Deviation				
	Peak Torque (Nm)	Pellet Size (µm)	AC Content (mg)	Release after 2 h (%)	Release after 8 h (%)
AC1	0.513 ± 0.022 (0.933) *	914 ± 47	14.85 ± 0.85	9.1 ± 0.84	100.00 ± 5.51
AC2	0.81 ± 0.047 (0.800)	1230 ± 87	13.95 ± 0.42	8.2 ± 0.54	71.45 ± 3.87
AC3	0.41 ± 0.021 (0.667)	789 ± 74	15.43 ± 0.53	7.79 ± 0.74	67.88 ± 4.21
AC4	0.534 ± 0.018 (0.667)	968 ± 41	15.62 ± 0.71	7.16 ± 0.41	84.42 ± 5.71
AC5	0.789 ± 0.071 (0.800)	1187 ± 35	14.23 ± 0.0.91	6.66 ± 0.45	78.52 ± 6.51
AC6	0.578 ± 0.054 (0.800)	990 ± 74	13.71 ± 0.62	6.22 ± 0.74	59.5 ± 4.60
AC7	0.498 ± 0.034 (0.933)	876 ± 58	15.81 ± 0.57	7.87 ± 0.85	92.78 ± 6.52
AC8	0.689 ± 0.047 (0.800)	1100 ± 71	13.95 ± 0.47	8.37 ± 0.98	98.7 ± 4.87
AC9	0.486 ± 0.021 (0.667)	823 ± 65	14.63 ± 0.79	7.32 ± 0.93	67.8 ± 3.45

* The number between parentheses refers to the binder ratio.

**Table 4:** Kinetic modeling of the release of AC from sustained-release matrix pellet formulations at pH 1.2 and pH 7.4.

Release Time	Formula	Zero-Order Model		First-Order Model		Higuchi Diffusion Model		Korsmeyer–Peppas Model	
		r	Slope	r	Slope	r	Slope	r	*n* *
0–2 h (pH 1.2)	AC1	0.957	0.073	0.961	−0.0003	0.998	0.842	0.992	0.539
	AC2	0.979	0.062	0.979	−0.0002	0.962	0.678	0.967	0.704
	AC3	0.979	0.059	0.979	−0.0002	0.962	0.644	0.967	0.704
	AC4	0.955	0.054	0.960	−0.0002	0.998	0.656	0.992	0.539
	AC5	0.944	0.050	0.960	−0.0002	0.998	0.610	0.992	0.539
	AC6	0.979	0.047	0.979	−0.0002	0.962	0.514	0.967	0.704
	AC7	0.957	0.062	0.960	−0.0002	0.998	0.720	0.992	0.539
	AC8	0.957	0.066	0.961	−0.0003	0.998	0.766	0.992	0.539
	AC9	0.979	0.055	0.979	−0.0002	0.962	0.605	0.967	0.704
2–8 h (pH 7.4)	AC1	0.975	0.226	0.801	−0.0073	0.940	4.667	0.946	1.196
	AC2	0.985	0.158	0.977	−0.0011	0.936	3.214	0.963	1.266
	AC3	0.985	0.150	0.979	−0.0010	0.936	3.053	0.963	1.266
	AC4	0.985	0.187	0.965	−0.0017	0.936	3.798	0.963	1.266
	AC5	0.987	0.173	0.974	−0.0014	0.940	3.528	0.968	1.220
	AC6	0.989	0.128	0.977	−0.0008	0.925	2.571	0.976	1.346
	AC7	0.985	0.205	0.942	−0.0023	0.936	4.174	0.963	1.266
	AC8	0.985	0.218	0.879	−0.0036	0.936	4.440	0.963	1.266
	AC9	0.986	0.154	0.978	−0.0010	0.917	3.376	0.974	1.347

r: correlation coefficient; n: release exponent. *: Obtained from the Korsmeyer–Peppas equation.

## Data Availability

The data presented in this study are available on request from the corresponding author.
